# Interdisciplinary Examination of the Communication Effect of Herbal Product Advertisements Targeting Health Problems in Older Adults: The Role of Advertising and Health Literacy

**DOI:** 10.1111/jep.70428

**Published:** 2026-03-30

**Authors:** Elçin Sebahat Kasapoğlu, Cihangir Kasapoğlu, Hanifi Dülger

**Affiliations:** ^1^ Department of Elderly Care, Vocational College of Health Services Bartın University Bartın Turkey; ^2^ Department of Marketing and Advertising, Vocational School Bartın University Bartın Turkey; ^3^ Department of Midwifery, Health Sciences Faculty Bartın University Bartın Turkey

**Keywords:** advertising literacy, health communication, health literacy, interdisciplinary, older adults

## Abstract

**Aim:**

This study examines how health literacy and advertising literacy influence older adults' responses to herbal product advertisements that claim to solve health problems. Older adults (OAs) are increasingly exposed to misleading health‐related messages through mass media, which may lead them to use unregulated and scientifically unproven products.

**Methods:**

A mixed‐methods explanatory sequential design was used with 237 older adults aged 65 years and over. In the quantitative phase, demographic data along with health literacy and advertising literacy scores were collected. Participants then viewed advertisements containing health claims. In the qualitative phase, their recall of ad content, attitudes toward the claims, and purchase intentions were assessed. Descriptive, comparative, and correlational analyses were performed; open‐ended responses were evaluated with thematic analysis.

**Results:**

OAs' health literacy was at a problematic–limited level (25.22 ± 8.77), whereas advertising literacy was moderate (91.07 ± 10.72). Higher health literacy was associated with more negative attitudes toward health claims (*p* = 0.004). Higher advertising literacy was positively associated with purchase intention (*p* < 0.001). Qualitatively, the most frequently recalled element was the health claim, and the most frequently mentioned word was pain.

**Conclusions:**

As health literacy improves, older adults become more skeptical of health‐claim advertisements; however, persuasive ad elements may still evoke purchase intentions. These findings underscore the need for interventions to enhance advertising and health literacy to protect elderly health.

## 1 Introduction

Population aging is accelerating worldwide, with the number of individuals aged 60+ expected to double by 2050 [1]. In Turkey, the proportion of OAs is projected to reach 11% by 2025 and 25.6% by 2080 [2], with aging, chronic diseases, functional decline, and increased healthcare utilization.

Older adults are among the heaviest consumers of traditional media. In Turkey, individuals aged 55+ watch the most television [3]. During the COVID‐19 pandemic, OA media use increased further [4]. Unregulated herbal product advertisements pose significant public health risks through systematically misleading marketing practices that exploit consumer trust. Multiple studies reveal critical vulnerabilities in health advertising. Specifically, 67.7% of consumers recognize testimonials as unreliable, and only 44.8% can distinguish genuine reviews [5]. Herbal supplements, largely unregulated by the FDA, are frequently marketed as safe despite potential contamination and adverse effects [6]. Although national broadcasters have removed some of these ads, similar content persists online and on foreign‐based satellite channels.

Ji et al. found that older adults' perceptions of cognitive aging, health anxiety, and loneliness significantly influence their irrational consumption tendencies [7]. Low health literacy substantially increases an individual's vulnerability by compromising their ability to understand, access, and use health information effectively. Multiple studies substantiate this claim. Talha Uçar et al. found that 59.6% of participants had either “insufficient” or “problematic‐limited” health literacy [8]. Çopurlar and Kartal et al. note that low health literacy negatively affects diagnosis and treatment processes, increases hospital admissions, and inappropriately burdens healthcare systems [9].

Research on advertising literacy among older adults remains critically limited, with most studies focusing exclusively on children and young adults. The existing literature reveals significant gaps in understanding how older populations process and critically evaluate advertising. Čábyová et al. specifically note that older adults may have an impaired ability to understand new forms of digital advertising, making them a vulnerable target group [10]. Bonifield and Cole further confirm that task characteristics and individual differences significantly affect older consumers' comprehension of persuasive communications [11]. An and Muturi provide one of the few targeted studies, demonstrating that older adults with low health literacy evaluate the value of educational advertising significantly lower than those with high literacy [12]. However, no comprehensive studies have yet examined both health and advertising literacy in older populations, a crucial research gap that warrants immediate scholarly attention. This study addresses this gap by examining how these two forms of literacy shape OAs' attitudes toward health claims and their purchase intentions. From a nursing perspective, particularly within internal medicine, older adults' exposure to misleading health‐related advertising represents a critical yet underexplored determinant of medication adherence, self‐management behaviors, and patient safety. Understanding older adults' health and advertising literacy is therefore essential not only for ethical advertising practices but also for nurses' roles in health education, risk communication, and the prevention of inappropriate herbal product use in chronic disease management.

### 1.1 Hypotheses

H1: Higher health literacy is associated with more negative attitudes toward herbal product ads containing health claims.

H2: Higher advertising literacy is associated with greater awareness and critical evaluation of such claims.

H3: Health and advertising literacy interact to influence attitudes toward ads and purchase intention.

## 2 Materials and Methods

### 2.1 Study Design and Participants

A mixed‐methods explanatory sequential design was used. Quantitative data were collected first to measure literacy levels and attitudes; qualitative data followed to enrich interpretation.

According to TURKSTAT, there were 31,348 OAs in a province in northwest Turkey in 2021. The study sample size was determined to be 195 participants, with a 95% confidence level and a 5% margin of error. Ultimately, 237 participants volunteered for the study. Eligible participants were individuals aged 65 and over. Data were collected from individuals aged older adults aged 65 years and over via face‐to‐face or video calls between October 2022 and February 2023. Participants were recruited using a snowball sampling method and were informed about the study objectives by the researchers. Data were collected from older adults who agreed to participate in the study.

Inclusion criteria included being an older adult aged 65 and over and agreeing to participate. Exclusion criteria included having serious vision, hearing, or speech problems and having a diagnosed psychiatric disorder. Older individuals who meet these criteria may have difficulty completing the survey.

### 2.2 Instruments

#### 2.2.1 The Türkiye Health Literacy Scale (THLS)

The scale was used to assess participants' health literacy levels. The THLS consists of 32 items. It has two dimensions: ‘Treatment and Service’ and ‘Disease Prevention and Health Promotion.’ Each dimension has four components: 1. Access to Health Information, 2. Understanding Health Information, 3. Evaluating Health Information, and 4. Using/Application of Health Information. The validity and reliability study in Türkiye was conducted by Okyay and colleagues [13]. The items on the Likert‐type scale are 1 = *very easy*, 2 = *easy*, 3 = *difficult*, 4 = *very difficult*, and 5 = *I have no idea*. The formula used to calculate the scale is: Index = (mean‐1) × (50/30). In this formula, the index represents the index calculated specifically for each individual, and the mean represents the average of each item answered by that person. In the scale assessment, 0 indicates the lowest health literacy, and 50 indicates the highest. As a result of the scoring, health literacy is expressed as inadequate if it falls between (0–25), problematic or limited if it falls between (> 25–33), sufficient if it falls between (> 33–42), and excellent if it falls between (> 42–50). Cronbach's alpha coefficient in the original study of the scale was 0.927. For this study, the internal consistency (Cronbach's alpha) was 0.934.

### 2.3 The Advertising Literacy Scale (ALS)

It was developed by Kömür and Boyraz based on an extensive literature review and is structured as a 5‐point Likert‐type instrument. The initial version of the scale consisted of 40 items; however, following an exploratory factor analysis, the final scale was reduced to 37 items and organized into six factors. These factors are awareness, following advertisements, benefiting from advertisements, obtaining information, interest in ads, and opposition to advertising. Reliability analyses indicated that the overall Cronbach's alpha for the scale was *α* = 0.74. The internal consistency coefficients for the subscales were as follows: awareness (*α* = 0.91), following advertisements (*α* = 0.86), benefiting from advertisements (*α* = 0.81), obtaining information (*α* = 0.78), interest in advertisements (α = 0.76), and opposition to advertising (α = 0.65). These results demonstrate that the scale has acceptable reliability [14].

### 2.4 The Attitude Toward Health Claims in Advertising Scale

The scale was developed by Atar. Attitudes toward health‐related claims in advertising were assessed using the items adopted from Atar's 2015 study. In this measurement approach, participants evaluate the clarity, informativeness, attention‐drawing nature, persuasiveness, sincerity, accuracy and misleading nature of the health claim presented in the advertisement. The items are rated on a 5‐point Likert‐type scale ranging from ‘Strongly disagree’ to ‘Strongly agree,’ with higher scores indicating a more positive attitude toward the health claim. This set of items is not a standardized standalone scale but is derived from Atar's original work examining consumer responses to health‐related advertising claims [15].

### 2.5 Procedures

#### 2.5.1 The Research Was Conducted in Two Stages

In the first stage, demographic data were collected from participants, including gender, age, education level, marital status, childbearing status, presence of chronic diseases, current medication use, and television viewing frequency. Additionally, health literacy and advertising literacy levels were assessed using the following scales.

In the second stage, participants viewed compiled advertisement videos featuring various herbal product brands marketed as medicinal solutions in paste, gel, and tablet forms for multiple health issues. Selected videos were previously aired on national TV channels but were banned from broadcast by the Radio and Television Supreme Council (RTSC). The study measured the impact of the advertisements on participants, focusing on recall of the claims, attitudes toward those claims, and purchase intentions. Following Atar's [15] suggestion, participants' recall of advertising claims was assessed using the ‘Attitude Scale Towards Health Claim in Advertising’ and ‘Intention to Buy Scale.’ Recalling the advertisement claim assumes it leaves a mental impression on viewers. In this method, participants are shown ads and asked if they noticed specific elements. In this study, participants were asked an open‐ended question about what they remembered after viewing the ad. Brand names in the video were concealed to prevent potential bias in participants' responses.

### 2.6 Data Analysis

Data collected from the personal information form and assessment scales were analyzed using SPSS software. Descriptive statistics such as frequency, percentage, mean, and standard deviation were computed. The chi‐square test was applied to categorical variables. The normality of the data was assessed using the Kolmogorov–Smirnov and Shapiro–Wilk tests for data sets that exhibited a normal distribution; independent *t*‐tests and One‐Way ANOVA were used for independent groups and dependent groups, respectively. For data that did not follow a normal distribution, the Mann–Whitney *U* test was utilized for independent groups, while the Wilcoxon signed‐rank test was used for dependent groups. A significance threshold of *p* < 0.05 was set. Following viewing the advertisement video, thematic analysis, a content analysis method, was employed to analyze participants' responses to the open‐ended question regarding remembered elements. Themes were derived from OAs' expressions using Maxqda software analysis.

### 2.7 Ethical Consideration

The required approvals were secured from the university's ethics committee (No:2022‐SBB‐0410), ensuring confidentiality and informed consent. Volunteers were briefed on the study's purpose, data collection methods, and confidentiality measures before obtaining their written and verbal consent.

## 3 Results

A study found that among participants, 61.6% were women, most aged 65–69 (57.2%). A majority were married (61.2%), had primary education (42.6%), were retired (52.8%), and had three or more children (64.6%). Additionally, 66.7% had a chronic disease, 77.6% used regular medication, 73.8% used herbal products, 83.1% ignored advertisements, 35.4% doubted product benefits for health, 33.8% watched TV over 3 h daily, and 62.9% didn't use social media.

It was found that there were meaningful differences in health (*p* = 0.019) and advertising (*p* < 0.001) literacy levels according to the genders of the OAs. While the mean health literacy score of men was 27.08 ± 7.76, it was found that women were 24.36 ± 9.79. Similarly, it was determined that men's advertising literacy score averages (93.83 ± 9.04) were higher than those of women (88.31 ± 12.41, Table 1).

**Table 1:** The mean scores of the participants from some demographic characteristics and the scales used (*n* = 237).

			THLS	ALS	Attitude towards health claims in advertisements	Purchasing intention
Demographic features		*n*	Mean ± SD	Mean ± SD	Mean ± SD	Mean ± SD
Gender	Female	146	24.36 ± 9.79	88.31 ± 12.41	20.44 ± 5.82	9.76 ± 2.50
	Male	91	27.08 ± 7.76	93.83 ± 9.04	19.06 ± 6.26	10.08 ± 2.21
	Value of the test	*t*	−2.248	−3.676	1.722	−1.022
		*p*‐value	0.019*	< 0.001*	0.086*	0.308*
Age	Between 65–69	125	26.58 ± 8.49	91.12 ± 11.03	19.15 ± 6.02	9.88 ± 2.42
	Between 70–74	56	25.96 ± 9.01	91.23 ± 10.35	20.44 ± 5.72	9.87 ± 1.89
	Between 75–79	33	20.07 ± 8.62	92.33 ± 12.28	21.24 ± 6.49	10:39
	80% and above	23	25.33 ± 11.33	82.00 ± 13.04	20.86 ± 5.75	9.17 ± 3.28
	Value of the test	*F*	4.760	4.862	1.553	0.202
		*p*‐value	0.003**	0.003**	1.170**	0.322
Marital status	Married	145	26.81 ± 8.66	91.86 ± 10.51	19.35 ± 6.07	9.90 ± 2.25
	Single	25	29.37 ± 9.96	90.08 ± 13.13	19.88 ± 5.68	9.56 ± 2.80
	Death of his wife	67	20.89 ± 8.27	87.47 ± 12.59	21.19 ± 5.92	9.97 ± 2.57
	Value of the test	*F*	13.505	3.389	2.061	0.274
		*p*‐value	< 0.001**	0.035**	0.130**	0.761**
Educational status	Not literate	59	19.50 ± 8.23	85.83 ± 12.64	21.66 ± 5.86	9.83 ± 2.76
	Primary school	101	25.51 ± 8.21	91.16 ± 10.47	20.07 ± 6.07	9.96 ± 2.26
	Secondary school	18	27.45 ± 10.59	91.72 ± 7.63	18.05 ± 5.53	9.77 ± 1.92
	High school	20	28.75 ± 8.49	94.35 ± 11.95	17.35 ± 5.61	10.10 ± 2.71
	University and above	39	31.41 ± 7.33	92.89 ± 12.12	19.02 ± 5.93	9.71 ± 2.28
	Value of the test	*F*	13.627	3.687	2.899	0.127
		*p*‐value	< 0.001**	0.006**	0.023**	0.973**
Chronic diseases	Yes	158	24.15 ± 9.01	90.31 ± 11.90	20.24 ± 6.29	9.96 ± 2.37
	None	79	27.91 ± 8.97	90.67 ± 10.83	19.26 ± 5.41	9.73 ± 2.46
	Value of the test	*t*	−3.029	−0.222	1.176	0.688
		*p*‐value	0.003*	0.809*	0.241*	0.492*
Continuous drug use	Yes	184	24.41 ± 8.96	89.96 ± 12.04	19.84 ± 6.21	9.91 ± 2.41
	None	53	28.85 ± 9.03	92.07 ± 9.48	20.16 ± 5.35	9.77 ± 2.37
	Value of the test	*t*	−3.167	−1.176	−0.348	−0.941
		*p*‐value	0.002*	0.241*	0.728*	0.699*

Abbreviations: ALS, The Advertising Literacy Scale; THLS: The Türkiye Health Literacy Scale.

* Independent samples *t*‐test

** One‐way ANOVA

There is a significant statistical difference in the factors of health literacy level, gender, age, marital status, education level, existence of chronic illnesses, and ongoing use of medication (*p *< 0.005). THLS scores were significantly higher for individual participants, those aged 65 to 69, individuals with a university degree or higher, people without chronic illnesses, and older adults who did not use medication continuously (Table 1).

Alongside the gender factor, there is a statistically significant difference in advertising literacy levels related to age, marital status, and education level (*p* < 0.005). The participants who were married, aged 75–79, and those who completed high school exhibited notably higher ALS scores (see Table 1).

Their attitudes towards the health claim in the advertisement differ significantly only in the education level variable (*p* < 0.005). The illiterate older adults (OAs) showed a more positive attitude towards health claims in advertisements, as indicated in Table 1.

The correlation analysis revealed a negative relationship between health literacy and attitudes regarding health claims in advertising. Specifically, as individuals' health literacy levels increased, their positive attitudes toward health claims in advertisements tended to decrease (*p* = 0.004). The study found a positive relationship between advertising literacy and purchase intention scales. It was determined that as the ad literacy levels of the participants increased, their purchasing intentions also increased (*p* < 0.001). Furthermore, it was observed that there is a positive relationship between the health claim score in the advertisement and consumers' purchasing intentions. Specifically, as the health claim scores in the ad rose, the likelihood of individuals intending to make a purchase also increased (*p* < 0.001, Table 2).

**Table 2:** Relationship and correlation coefficients between advertising literacy and health literacy.

		THLS	ALS	Attitude towards health claims in advertisements	Purchasing intention
THLS	rp‐value	1			
ALS	rp‐value	0.121* 0.063	1		
Attitude towards health claims in advertisements	rp‐value	−0.184* 0.004	0.026* 0.690	1	
Purchasing intention	rp‐value	0.134* 0.039	0.301* < 0.001	0.440 < 0.001	1

Abbreviations: ALS, The Advertising Literacy Scale; THLS, The Türkiye Health Literacy Scale.

* Pearson Correlation is significant at the 0.001 level (2‐tailed).

**Figure 1:**
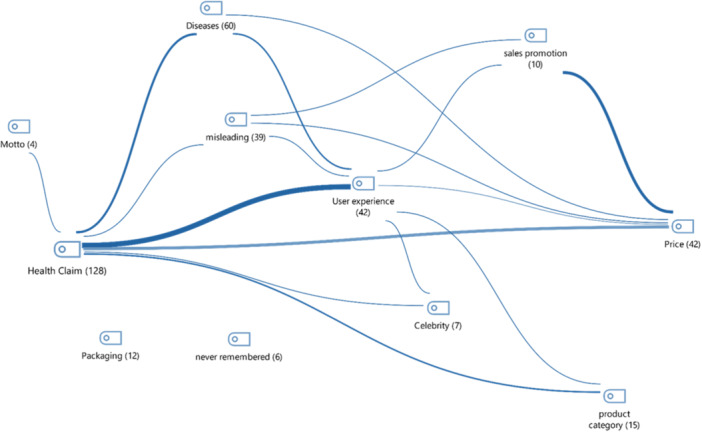
Co‐occurrence map of themes remembered in the advertisement.

OAs were asked what they remembered about the commercial video as an open‐ended question. The data were coded according to the expressions of the OAs and themes were created. In this context, the themes and frequencies created according to the answers are: health claim(128), diseases(60), user experiences(42), price(42), misleading(39), product category(15), packaging(12), diseases(10), sales promotion(10), famous person(7), not remembering anything(6) and motto(4). Accordingly, participants mostly remembered the health claim given in the advertisements. In Figure 1, the themes are organized based on how often they occur together. This suggests that the user experience is often recalled alongside the primary health claims presented in the advertisement viewed. In the advertisements, the ‘experiences of those who use the product’ and the ‘price’ of the products were the second most remembered elements. The themes of packaging and not remembering anything did not occur in conjunction with any theme (Figure 1).

‘What elements of the ad do you remember?’ The most used and remembered concept in the answers to the question is the idea of ‘Pain.’ The concept of pain is followed by the concepts of ‘low back’ and ‘hernia.’

## 4 Discussion

Older adults (OAs) are increasingly exposed to health product advertisements on television and social media, and this exposure may disproportionately affect those with chronic conditions who tend to believe health claims. Limited understanding of health information further exacerbates this issue, potentially increasing inappropriate product use and avoidable hospital or emergency room visits, thereby posing a significant public health concern. Despite growing discussions around misleading health‐related advertisements, no studies have specifically examined the relationship between harmful advertising and the expanding OA population globally or in Turkey. This study contributes to filling this gap by examining how health claim advertisements influence OAs and how these influences relate to their health literacy levels, underscoring the need to improve both health and advertising literacy to protect public health.

Consistent with previous research, our study found that OAs generally have insufficient or limited health literacy [16, 17, 18]. Although many existing studies examining literacy focus primarily on children and young populations [19, 20], research conducted with adults in Turkey reported moderate levels of media literacy [21]. Similarly, Atar found that OAs particularly those aged 48 and older, married, and with children show moderate interest in health claims in advertisements and demonstrate comparable purchase intentions [15].

Although most participants in our study were women, male OAs exhibited significantly higher health and advertising literacy levels. However, gender did not influence attitudes toward health claims or purchase intentions. These findings are consistent with a Dutch study reporting higher health literacy among older men [16], yet contrast with studies conducted in Iran showing higher health literacy among older women [17, 18]. Cultural and social differences across countries may help explain these variations.

Health literacy was higher among OAs aged 65–69, single individuals, and those with university or higher education, whereas advertising literacy was higher among married OAs aged 75–79 with high school diplomas. Prior research shows that advertisements can mislead individuals across all age groups through persuasive communication strategies [22], highlighting the importance of skills that enable people to recognize and respond to deceptive messages [23]. Since health literacy tends to be lower among OAs compared with younger age groups [24, 25], individuals with low literacy may be more vulnerable to misleading advertisements promoting unverified benefits for chronic diseases. This underscores the importance of digital literacy training to support healthier habits and improve overall quality of life [26].

Improving OAs' ability to critically navigate digital advertisements is essential for enhancing their overall well‐being, and this requires strengthening both health and advertising literacy [27, 28, 29]. When designing health behavior interventions for older individuals, considering factors such as social capital and e‐health literacy is also crucial [30]. Low health literacy can hinder patients' understanding of health information and limit their ability to seek appropriate medical care [29]. Disseminating health information through community networks may help reduce the negative impacts of limited literacy [31]. Additionally, consistent with prior research, illiterate OAs in our study showed lower health and advertising literacy levels and were more susceptible to false health claims [32, 33].

Health literacy was significantly lower among OAs with chronic diseases and those regularly using medications. Prior studies similarly show that individuals over 65 with chronic illnesses, low education, or limited functional capacity tend to have lower health literacy levels [34]. These findings reinforce the need to enhance literacy through social support and community‐based networks.

Our study found no correlation between OAs' health literacy and their ability to understand health claims in advertisements. However, previous research in Turkey identified a moderate positive association between health literacy and media literacy [21]. Literacy skills enable individuals to make informed decisions regarding health, diet, and lifestyle [32, 33]. Access to reliable health information also improves self‐management among individuals with chronic illnesses [31]. Communication‐based literacy interventions and health education play key roles in social marketing and health promotion [35]. To combat misleading herbal product advertisements, public campaigns warning consumers about deceptive health claims may be beneficial.

Participants with higher health literacy were able to identify unrealistic aspects of the health claim advertisements they viewed; however, advertising literacy did not significantly influence their attitudes toward these claims. Notably, even those with high literacy levels reported an intention to purchase advertised products, suggesting that persuasive techniques and perceived low financial risk may outweigh critical evaluation. These findings highlight the need for stricter regulatory measures, as misleading advertisements persist through satellite channels and social media despite removal from national broadcasts by the RTSC. Advertisers should also enhance their own health literacy and adhere to ethical standards to prevent misleading communication.

Despite negative attitudes toward advertisements and higher health literacy, some OAs still intended to purchase these products, possibly driven by hope for symptom relief. Although advertising literacy positively correlated with purchase intention, it did not influence attitudes toward health claims, suggesting that emotional and experiential factors may be more influential than cognitive evaluation.

In the open‐ended responses, most participants recalled product benefits, pain‐related claims, and price information. The limited recall of celebrities suggests that celebrity endorsements may be less influential among OAs than expected. These qualitative insights enrich the interpretation of the quantitative findings by highlighting which aspects of advertisements resonate most with this demographic.

Future research should focus on developing advertising and health literacy interventions tailored to OAs, as most existing programs target children and younger populations [20, 36]. Given the increasing presence of direct‐to‐consumer health advertisements [37] and widespread misinformation in digital media [4], strengthening OAs' ability to critically evaluate health‐related messages is essential. By addressing the limited literature on health and advertising literacy among OAs, this study provides valuable insights to guide health communication strategies aimed at supporting chronic disease management and enhancing public health protection through responsible use of advertising and social media.

### 4.1 Strengths and Limitations

The primary strength of this study is the adoption of an Explanatory Sequential Mixed Methods Design. This design provides methodological strength to the study. Quantitative data (health and advertising literacy levels) were collected first, followed by qualitative data to provide deeper context for the findings and enrich the interpretations. A second strength of the study is its focus on older adults aged 65 and over, a vulnerable sample. The results of this study, conducted with this challenging sample group, fill an important gap in the field. Furthermore, the use of real commercial videos, prohibited by the RTSC, as stimuli and the concealment of brand names to prevent potential bias enhanced ecological validity.

Limitations of this study include that participants were recruited from only one province in Northwest Turkey, and the findings may not be representative of the entire older adult population nationwide. The fact that the advertising videos used in the second phase were previously banned may have made them less representative of the current advertising landscape and potentially have a different impact on participants than usual.

### 4.2 Implications for Practice

The findings of this study could inform strategies for health professionals, particularly nurses, in their public health and chronic disease management training programs, incorporating knowledge and awareness about products found in such digital media. They also offer critical practical implications for advertising regulators. Nurses, particularly those working in public health and internal medicine, should repurpose patient education materials for older adults with chronic illnesses and low health literacy into a simple, visually supported, and critically stimulating format. They should also actively advocate for herbal product advertisements regarding potential drug interactions and the risks of non‐adherence. Furthermore, professionals in advertising and marketing, recognizing older adults' high reliance on health claims and user experiences and the vulnerability associated with low literacy, should prioritize their ethical responsibilities and avoid misleading content. Finally, regulatory bodies (RTSC/Ministry of Health) should tighten their oversight of health claims and user stories, which are the most memorable elements of advertising and increase purchase intent, focusing particularly on preventative measures to protect at‐risk older adult populations.

## 5 Conclusion

The main findings of this study indicate that older adults have limited health and advertising literacy, and this is closely linked to demographic factors; women, those with low education, and those with chronic illnesses are particularly at risk. Another key finding of the study is that as health literacy increases, older adults' positive attitudes toward health claims in advertisements decrease significantly. However, the health claim and user experiences, which are the elements most remembered by older adults, positively increase purchase intentions even when advertising literacy is high. This suggests that the persuasive power of advertising messages has the potential to override literacy levels. This suggests the need to strengthen multidisciplinary strategies and partnerships to protect this sample group.

## Author Contributions

**Elçin Sebahat Kasapoğlu:** conceptualization, methodology, formal analysis, investigation, writing – original draft, writing – review and editing, writing – critical review, supervision. **Cihangir Kasapoğlu:** conceptualization, methodology, formal analysis, investigation, writing – original draft, writing – review and editing, writing – critical review, supervision. **Hanifi Dülger:** conceptualization, methodology, formal analysis, investigation, writing – original draft, writing – review and editing, writing – critical review, supervision. All listed authors meet the authorship criteria, and all authors are in agreement with the content of the manuscript.

## Funding

The authors received no specific funding for this work.

## Ethics Statement

The required approvals were secured from the university's ethics committee (No:2022‐SBB‐0410), ensuring confidentiality and informed consent. Volunteers were briefed on the study's purpose, data collection methods, and confidentiality measures before obtaining their written and verbal consent.

## Conflicts of Interest

The authors declare no conflicts of interest.

## Data Availability

The data that support the findings of this study are available from the corresponding author upon reasonable request.
